# Contributions to Urology

**Published:** 1861-01

**Authors:** William A. Hammond

**Affiliations:** Professor of Anatomy and Physiology in the University of Maryland, &c.


### SELECTIONS.

*Contributions to Urology.* By William A. Hammond, M.D., S. C., Professor of Anatomy and Physiology in the University of Maryland, &c.

It is a principle indissolubly connected with vitality in both plants and animals, that decomposition of the tissues constituting their several structures is incessantly taking place, either as a cause or consequence of the various organic actions they are called upon to perform. The products of this constant disintegration are incapable of entering into such combinations as the healthy organism requires for its nutrition, and although, under certain abnormal circumstances, some of them do contribute to the maintenance of functions necessary to life, they must, nevertheless, be regarded as essentially effete. Accordingly, we find that they are continually being removed to make way for newer and more vigorous material, and after in many instances undergoing further degradation, eventually fall to the inorganic class of substances whence they originally came.

With tissue-destruction, as it occurs in the vegetable kingdom of organic nature, it does not come within the scope of this memoir to treat; but a few of the main facts connected with its course in animals may very properly engage a portion of our attention.

It may be considered a fully established law, that the decay of the tissues of any organ of the system is in direct proportion to the functional activity of that organ. By the term decay, it is not intended to refer to atrophy or degeneration, which are inversely to the functional activity of a part, but to that interstitial death of tissues which always, in health, calls for increased deposition of new material, and may even produce hypertrophy.

We should, *a priori*, expect the existence of the law above enunciatied; for, even in the operation of artificial machines, we find that those parts involved in the greatest degree of action are soonest worn out. In the galvanic battery, for instance, the amount of dynamic electricity evolved is always in proportion to the quantity of matter subjected to chemical action, and, consequently, we are enabled to measure the *relative* amount of the one from the *absolute* amount of products resulting from the operation of the other. It must not be forgotten, however, that an essential distinction exists —in addition to the radical differences of composition and structure—between the parts of such machines and those of an organic being, in this, that in the latter there is a compensating deposit of new substances, whilst in the former no such action occurs.

Experiments are not, however, wanting to support the principle above stated. Increased muscular exertion—*cæteris paribus*—is invariably followed by an augmentation of the quantity of urea and other constituents of the urinary excretion, as has been determined by the researches of Simon,* Lehmann,† myself,‡ and others.‖ Excessive mental exertion, as is well known, induces and increased elimination of phosphoric acid through the kidneys, and some time since I attempted to show the relation existing between the amount of this substance excreted and the extent of mental exercise. Rummela§ and Draper,¶ it is true, arrive at conclusions which, so far as they relate to the increased elimination of urea as an effect of augmented physical exertion, are at variance with the above; but the great mass of authority is in accordance with the view above given.

It has also been ascertained, that the excretions from the skin and lungs are augmented by increased wear of these

* Animal Chemistry, Cavendish Society's edition, vol. 2, p. 168.
† Physiological Chemistry, Cavendish Society's edition, vol. 1, p. 163.
‡ American Journal of the Medical Sciences, January, 1853.
‖ North American Medico-Chirurgical Review, vol. 1, p. 730.
§ Transactions of the Royal Society of Edinburgh, vol. xxi, p. 543.
¶ American Journal of the Medical Sciences, No. lviii. Apri, 1855, p. 334.

parts of the organism. Numerous authorities might be adduced in support of this statement; but it is a fact so patent to every observer, that the simple allusion to it is sufficient.

Of the several channels which exist for the removal of decomposed tissue from the system, that of the kidneys is the only one which is purely and entirely excretive. The lungs perform the double office of eliminating carbon and absorbing oxygen; the skin gives off water, chlorides, phosphates, &c., and is also capable of taking up substances placed in contact with it; and the intestines serve for the absorption of nutriment, and for giving exit to unassimilated food, as well as for the evacuation of product of destructive metamorphosis. Thus, therefore, when we place a ligature around the trichea of an animal, death ensues from two causes; when the skin of a frog is covered with a varnish impermeable to air and moisture, the animal dies as well fsom obstruction to absorption as from arrest of elimination; and when the function of the intestines is stopped, death ensuos more from starvation than from the non-removal of effete matters from the organism; but if the renal arteries be tied, or the kidneys removed from an animal, death is essentially due to arrest of excretion, and the consequent retention in the blood of substances fatal to organic existence.

The urinary excretion, within the limits of health, is subject to great variation, both in quantity and constitution, from the operation of various disturbing causes. Thus the quantity of water may be influenced by the quality of the food, by medicines, and by the temperature and humidity of the atmosphere. The amount of solids dissolved in it is also subject to alteration from like causes; and, in short, any cause affecting the rate of retrogressive tissue metamorphosis, and, as a consequence, the quantity of products arising from tissue-decay, must modify, more or less, the character of the urine. When we reflect that every contraction of our muscles, every action of the various glands of our bodies, every thought of our brains—in fact, every functional operation, however short it may be—involves the destruction of a certain amount of tissue, we can well percieve how varied must be the composition of an excretion containing the representatives this decay. It is impossible, therefore, to assign any definite composition to the urine, so far as relates to the quantity of any of its component parts.

Various analyses of the normal human urine, showing its composition in 1,000 parts, have been made. As might be expected, no two of these are alike, and for all physiological or practical purposes, must be nearly or altogether useless. It is only by analyzing the entire quantity of urine vacuated in a given time, under known conditions of health, food, physical and mental exercise, &c., that any indications useful to science are likely to be obtained.

Great diversity exist in the composition and character of the urine as excreted by the several orders and classes of animals. In the invertebrates, we know but little of this fluild. In the excrements of certain insects, uric acid and urates have been discovered. I have found very beautiful crystals of uric acid in the excremental matters of certain larval springidæ, and also in those voided by several species of coleopterous insects.

In fish, the urine has been but little studied. Davy* found that a nitrogenous compound—either urea, a combination of uric acid, or some other azotized substance—was excreted.—Jones,† in the urine of the bass (*corrina ocellata*,) detected uric acid, oxalate of lime, phosphates, and chloride of sodium; and in that of the white fish (*corrigonus alba*), I have found all these substances, together with urea and sulphuric acid.

In reptiles, we meet with considerable diversity in the composition of the urine. In frogs, it contains urea in quite a large proportion, chloride of sodium, and phosphoric acid. In serpents, it is composed for the most part of urates, although under certain circumstances I have detected urea. In lizards, both urea and uric acid are found, altogether with

* Transactions of the Royal Society of Edinburgh, vol. xxi. p. 543.
† American Journal of the Medical Sciences, No. lviii. April, 1855 p. 334.

phosphoric acid in small amount. In the urine of *plectiodon obsoletum* the urea amounted to 25.00 of the total quantity of solids, and in that of *ophisaureus ventralis* to nearly 15.00. In *phrynozoma cornutum*, there was barely a trace of urea, but uric acid in large amount. In the chelonians—the urine of which I have examined—both urea and uric acid were found, together with hippuric, sulphuric, and phosphoric acids.

In birds, the urine consists almost entirely of uric acid in combination with ammonia and lime. In common with almost every other observer, I have never detected urea in the urine of this order of animals.

In the mammalia we meet with a more complex excretion. In the carnivora, however, it consists mainly of a solution of urea in water. The urine of a cat weighing 4¾ pounds, fed for several days on a diet composed exclusively of animal food, I found to consist as the average of five daily analyses of

| | |
|---|---|
| Water, | 90.32 cubic centimetres |
| Urea, | 9.50 grammes. |
| Inorganic salts, | 1.47 grammes. |

The urine of the herbivora is a far different fluid. Instead of being acid, it, always alkaline, contans no uric acid, but abounds in alkaline carbonates, and other substances not present in the urine of the animals last mentioned.

The urine of man is an exceedingly complex fluid. Being the most highly organized of all animals it is reasonable to suppose that this would be the case; for the more simple the structure of anorganism, the more simple in composition are its excretions, and as organization becomes more complicated as we ascend the scale of creation, the excretions, made up as they are, mainly of the detritus of organs, come to contain substances not present in those of less elaborately constructed beings.

The main purpose of the kidneys is, undoubtedly, to remove the nitrogenous products of tissue metamorphosis from the system. Such substances, we find, especially in man constitute a large proportion of the solid matters of the urine, amounting in many instances to more than 75.00 of the total quantity eliminated in the twenty-four hours. In addition, there are various substances belonging to the mineral kingdom.

The primary division of the constituents of the urine, would, therefore, be into the organic, and inorganic.

Under the head of organic substances are embraced:

1. Urea.
2. Uric acid free and in combination with lime, soda, magnesia and ammonia.
3. Hippuric acid in combination with soda and perhaps other bases.
4. Creatine.
5. Ceartinine.
6. Oxalic combined with lime.
7. Coloring matters.

The inorganic constituents of the normal human urine are as follows:

1. Water.
2. Chlorine combined with sodium, potassium and ammonium.
3. Sulphuric acid in combination with lime and potash.
4. Phosphoric acid in combination with lime, magnesia, potash, soda and ammonia.
5. Carbonic with lime.

The average amount of urine excreted in the twenty-four hours by adults in good health, taking a moderate amount of exercise, and eating a due proportion of animal and vegetable food, I have found, as the result of numerous determinations, to amount to from 900 to 1600 c. cm. The solids in the same period, and under like conditions, ranged from 30 to 110 grammes. In ten healthy adult males, weighing each about 165 pounds, being from 23 to 15 years of age, and living under similar conditions of food, exercise, &c., I obtained as the average of ten examinations in each individual case, the following:

Whole quantity of urine, 1102.11 cubic centimetres.
Solids, 55.46 grammes.

We see, therefore, how great is the amount of drainage from the system through this channel, and we can readily perceive how important it is, in sanitary point of view, that it should continue uninterrupted.

The modifications which the urine undergoes, in health and disease, I purpose considering in a future paper.—*Maryland & Virgina Medical Journal.*

